# The thermogenic characteristics of adipocytes are dependent on the regulation of iron homeostasis

**DOI:** 10.1016/j.jbc.2021.100452

**Published:** 2021-02-23

**Authors:** Jin-Seon Yook, Mikyoung You, Yongeun Kim, Mi Zhou, Zhenhua Liu, Young-Cheul Kim, Jaekwon Lee, Soonkyu Chung

**Affiliations:** 1 Department of Nutrition and Health Sciences, University of Massachusetts, Amherst, Massachusetts, USA; 2 Department of Nutrition and Health Sciences, University of Nebraska, Lincoln, Nebraska, USA; 3 Department of Biochemistry, University of Nebraska, Lincoln, Nebraska, USA

**Keywords:** iron regulatory proteins, iron-response element, uncoupling protein, thermogenesis, brown fat, adipose iron, mitochondria, iron homeostasis, labile iron pool, AdipoQ, adiponectin, CytC, cytochrome C, DFO, deferoxamine, ER, endoplasmic reticulum, eWAT, epididymal white fat, FBXL5, iron-stabilized E3 ligase component, Fe-S, iron–sulfur cluster, Fe-Tf, transferrin-bound iron, FT, ferritin, FTL, ferritin light chain, *h*SV, human stromal vascular cells, iBAT, interscapular brown fat, IRE, iron-response element, IRP, iron regulatory proteins, ISCU, iron–sulfur cluster scaffold protein, iWAT, inguinal white adipose tissue, LIP, labile iron pool, OxPhos, oxidative phosphorylation, PGC1α, Peroxisome proliferator-activated receptor-gamma coactivator alpha, PPARγ, peroxisome proliferator-activated receptor-gamma, PRDM16, PR-domain containing 16, TfR1, transferrin receptor 1, UCP1, uncoupling protein 1

## Abstract

The development of thermogenic adipocytes concurs with mitochondrial biogenesis, an iron-dependent pathway. Iron regulatory proteins (IRP) 1 and 2 are RNA-binding proteins that regulate intracellular iron homeostasis. IRPs bind to the iron-response element (IRE) of their target mRNAs, balancing iron uptake and deposition at the posttranscriptional levels. However, IRP/IRE-dependent iron regulation in adipocytes is largely unknown. We hypothesized that iron demands are higher in brown/beige adipocytes than white adipocytes to maintain the thermogenic mitochondrial capacity. To test this hypothesis, we investigated the IRP/IRE regulatory system in different depots of adipose tissue. Our results revealed that 1) IRP/IRE interaction was increased in proportional to the thermogenic function of the adipose depot, 2) adipose iron content was increased in adipose tissue browning upon β3-adrenoceptor stimulation, while decreased in thermoneutral conditions, and 3) modulation of iron content was linked with mitochondrial biogenesis. Moreover, the iron requirement was higher in HIB1B brown adipocytes than 3T3-L1 white adipocytes during differentiation. The reduction of the labile iron pool (LIP) suppressed the differentiation of brown/beige adipocytes and mitochondrial biogenesis. Using the ^59^Fe-Tf, we also demonstrated that thermogenic stimuli triggered cell-autonomous iron uptake and mitochondrial compartmentalization as well as enhanced mitochondrial respiration. Collectively, our work demonstrated that IRP/IRE signaling and subsequent adaptation in iron metabolism are a critical determinant for the thermogenic function of adipocytes.

At least three different types of adipocytes exist in mammals based on thermogenic capacity, nonthermogenic white adipocytes and two types of thermogenic adipocytes, *i.e.*, constitutively brown and inducible beige adipocytes (1). White adipocytes are specialized to store extra energy into triglyceride (TG), while brown/beige adipocytes are proficient in burning energy into heat (2). White adipocytes have a big and unilocular lipid droplet to maximize the triglyceride storage capacity, while brown/beige adipocytes possess small and multilocular lipid droplets to facilitate beta-oxidation (2). In addition to these morphological features, the brownish color of adipose tissue provides an immediate clue to distinguish the thermogenic capacity of the adipose depots. The brownish tint of adipocytes proportionally increases with the number of mitochondria and uncoupling protein 1 (UCP1). More precisely, the brownish color of thermogenic adipocytes stems from the iron–porphyrin complexes of the mitochondrial respiration chain (3). Moreover, nonheme iron found in the iron–sulfur cluster (Fe-S) proteins plays a crucial role in the redox reaction of electron transfer in mitochondria. Although these studies suggest that iron demands for brown/beige adipocytes are higher than that of white adipocytes, little information is available regarding the iron regulatory mechanisms that discern white, beige, and brown adipocytes.

The depot specificity of adipose tissue is dependent on the developmental and transcriptional traits of the progenitor cells. It has been demonstrated that white and beige precursor cells share the white adipocyte lineage, while classical brown precursor cells share their ancestral lineage with myocytes (4). Recently, the existence of four distinct adipocyte progenitors is identified in human mesenchymal stem cells that differentiate into different adipocyte subtypes (5). Intriguingly, one of the key characteristics to discern thermogenic cell progenitors from white adipocyte precursors is the transcriptional capacity accumulating iron and protecting oxidative stress (5). These results implicate that the modification of iron metabolism constitutes the fundamental processes involving thermogenic adipocyte formation.

The cellular iron metabolism is tightly regulated by the intracellular need of iron to coordinate iron uptake, storage, and export *via* iron regulatory proteins (IRP) 1 and 2 (6). IRP1 and 2 are RNA-binding proteins that regulate iron metabolism-related gene expression in posttranscriptional levels. In iron adequate conditions, IRPs are unable to bind to iron-responsive elements (IREs). The Fe-S cluster binding-induced conformational change in IRP1 blocks its interaction with IREs at its target RNA species, and thus IPR1 mainly serves as cytosolic aconitase. IRP2 undergoes rapid proteasomal degradation in the presence of sufficient iron (6). In iron-depleted conditions, IRPs bind to IREs at the 5′ or 3′ UTR of target mRNAs encoding proteins involved in iron uptake, storage, and export; The IRP-IRE binding at the 3′ UTR region in transferrin receptor 1 (*Tfr1*) increases RNA stability, promoting iron uptake. In contrast, IRP-IRE binding at the 5′ UTR region in ferritin (*Ft*), an iron storage protein, represses its translation, which decreases iron sequestration but increases intracellular labile iron pool (LIP) (6) (Fig. 1*A*).

**Figure 1:**
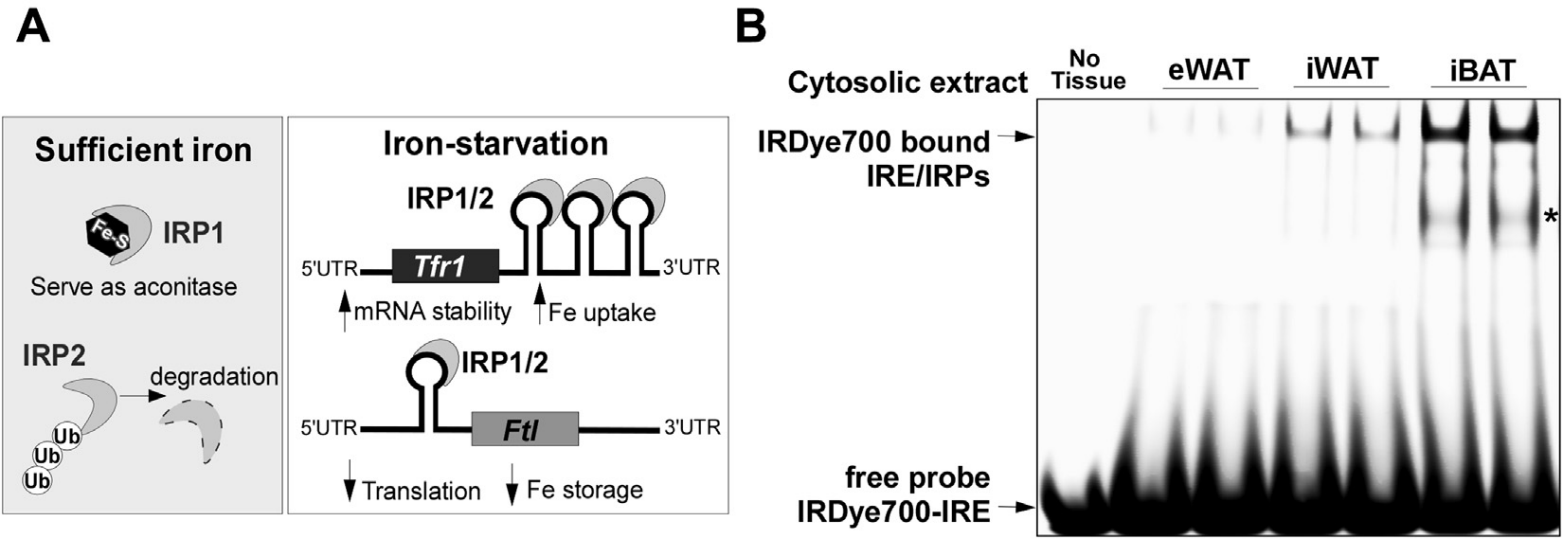
**IRP binding to IRE was differentially regulated in white *versus* brown adipose tissue.***A*, schematic drawing for the regulation of IRP1 and IRP2 in response to iron availability. Under iron-sufficient conditions, IRP1 serves as cytosolic aconitase, and IRP2 undergoes proteasomal degradation. In contrast, both IRP1 and IRP2 bind to IREs of their target genes, including transferrin receptor 1 (*Tfr1*) and ferritin (*Ft*) in iron-starvation conditions. IRP/IRE binding promotes iron uptake by stabilizing *Tfr1* mRNA and decreases iron storage by inhibiting the translation of ferritin. *B*, electrophoretic mobility shift assay (EMSA) by using IRDye700-IRE probe. The cytosolic fractions were prepared from epididymal (eWAT), inguinal white adipose tissue (iWAT), and interscapular brown fat (iBAT). ∗ denotes a nonspecific binding. The image is a representative of three different experiments.

Despite the well-established posttranscriptional regulation that cells have evolved to maintain the iron homeostasis against the fluctuating environmental iron, little is known about how this IRP/IRE regulatory system plays a role in the development of different adipocyte subtypes. In this study, we aimed to investigate the differential regulation of iron metabolism in white, brown, and beige adipogenesis by using the depot-specific adipose tissue as well as defined cell models of white, beige, and brown adipocytes. Our results demonstrate that enhanced IRP/IRE interaction and the subsequent iron influx into the mitochondria are essential for the differentiation of thermogenic brown and beige adipocytes.

## Results

### IRP/IRE signaling and iron metabolism in adipose tissue modulate the thermogenic potential

**Figure 2:**
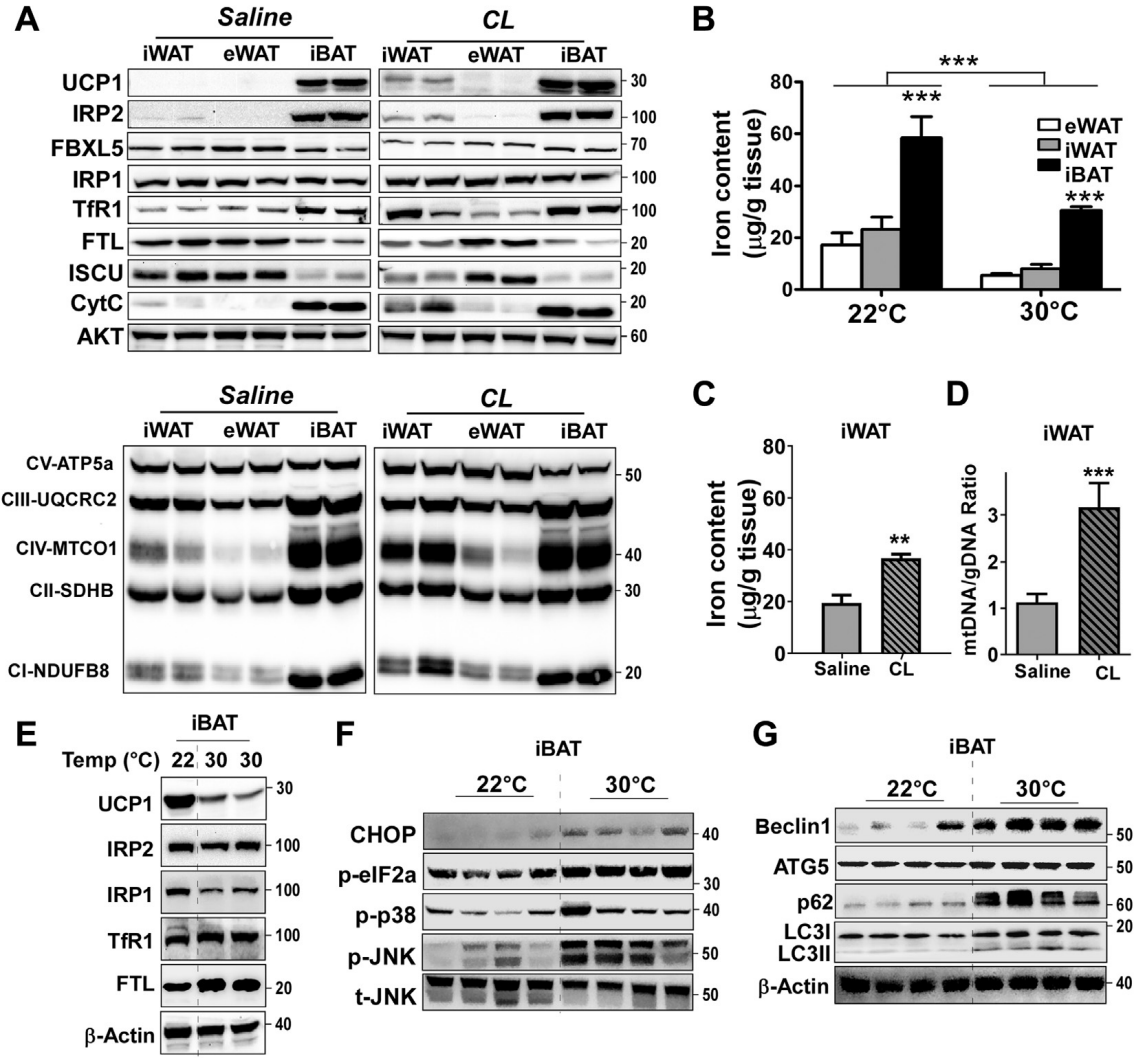
**The iron content of adipose tissue was modulated to meet the cellular demands for thermogenesis and mitochondrial biogenesis.** C57BL/6 mice were administrated with either saline or β3-adrenoceptor agonist (CL) for 5 days (n = 8/group), and the epididymal (eWAT), inguinal (iWAT), and interscapular (iBAT) fat were collected (*A*–*D*). Or, adipose depots were collected from the mice kept in either normal physiological (22 °C) or thermoneutral temperature (30 °C) for 14 days (n = 8/group) (*E*–*G*). *A*, the changes of proteins related to iron handling (*upper*) and mitochondrial respiratory chain components (*lower*, OxPhos protein cocktail from Abcam was used), *B*, iron content of depot-specific adipose tissues kept in either 22 °C or 30 °C, *C*, iron content of iWAT with or without CL treatment for 5 days, *D*, the ratio of mitochondrial DNA to genomic DNA in iWAT with or without CL treatment, *E*, the protein expression related to iron metabolism and UCP1 from iBAT kept in either 22 °C or 30 °C, *F*, protein analysis related with ER stress from iBAT kept in either 22 °C or 30 °C, *G*, autophagy-related protein expressions from iBAT kept in either 22 °C or 30 °C. All western blot images represent three separate images, and each lane (loaded 25 μg protein) stands for tissue samples from an individual animal. Different loading controls were used, including AKT (in *A*), t-JNK (in *F*), and β-Actin (in *E* and *G*). Data in *B*–*D* are presented as mean ± SD. In *B*, data are analyzed by two-way ANOVA (adipose depot and temperature) ∗∗∗*p* < 0.001 (n = 6/group). In *C* and *D*, ∗∗*p* < 0.01, ∗∗∗*p* < 0.001 by one-way ANOVA (n = 5/group).

Cellular iron homeostasis is tightly regulated by two cytoplasmic RNA-binding proteins, iron regulatory protein (IRP) 1 and 2 (Fig. 1*A*). IRPs/IRE regulatory system is likely operated in a cell-type-specific manner to satisfy metabolic needs. Based on the high metabolic rate of brown fat, we first hypothesized that iron demands and IRP/IRE interaction would be higher in brown adipose tissue than white adipose tissue. To investigate IRP/IRE binding, we performed an electrophoretic mobility shift assay (EMSA) using an IRDye700-labeled IRE probe with the cytosolic fractions prepared from epididymal (eWAT), inguinal white adipose tissue (iWAT), and interscapular brown fat (iBAT) that represent visceral, subcutaneous fat and classical brown fat, respectively. The IRP/IRE binding was barely noticeable, and only free IRE was observed in eWAT, while IRP/IRE binding was obvious in iWAT. In contrast to WAT, the IRP/IRE band was intense in the cytosolic fraction of iBAT, indicating the higher demands for iron in BAT than WAT (Fig. 1*B*). We next examined the expression levels of iron-handling proteins and mitochondrial respiratory components in each depot. In the basal levels, iron-deficiency responses (*i.e.*, IRP2 accumulation, increased TfR1, and reduced FT light chain), were apparent in iBAT, but not in eWAT and iWAT (Fig. 2*A*, *Left*), which is consistent with the intense IRP/IRE-binding pattern (Fig. 1*A*). Notably, the endogenous levels of Fe-S cluster scaffold protein ISCU were distinctively lower in iBAT compared with iWAT or eWAT (Fig. 2*A*). The expression levels of the respiratory chain components were significantly higher in the iBAT than eWAT or iWAT, which is consistent with more mitochondrial OxPhos proteins in iBAT than WAT (7). Reflecting the increased TfR1 protein expression in iBAT, the total iron content in iBAT was approximately threefold higher than in eWAT or iWAT (Fig. 2*B*).

Next, we questioned whether the environmental stresses that alter the thermogenic potential could also modulate the intracellular iron content. To test this, we administrated the C57BL/6 mice with β3-adrenergic agonist CL 316,243 (CL) for 5 days to induce adipose tissue browning, and depot-specific adipose tissues were collected. In agreement with the fact that eWAT is less susceptible to adipose browning than iWAT, CL treatment did not affect the expression levels of UCP1 or IRP2 in eWAT. Importantly, enhanced browning in iWAT was accompanied by a significant increase of iron-starvation responses, including stabilized IRP2, increased TfR1, and decreased FTL and ISCU expression along with increased UCP1 expression (Fig. 2*A*, *Right*). Consistently, the iron content in iWAT was increased in response to CL treatment (Fig. 2*C*), with the rank order of iron content as eWAT < iWAT < iWAT_CL_ < iBAT. The increase of iron content in response to CL treatment was consistent with increased mitochondrial respiratory chain proteins (Fig. 2*A*, *Right*) and mitochondrial DNA content (Fig. 2*D*). Conversely, when the mice were kept in thermoneutral conditions (30 °C) for 2 weeks to dampen the thermogenesis, the iron content of adipose tissue was significantly reduced compared with iron levels at ambient temperature (22 °C) in all three adipose depots (*p* < 0.001) (Fig. 2*B*). Within the same temperature setting, iBAT maintained the highest iron content when compared with eWAT and iWAT (*p* < 0.001) (Fig. 2*B*). The decrease of UCP1 levels of iBAT at 30 °C induced an increase of FTL, suggesting an iron deposition into the cytoplasm (Fig. 2*E*). Intriguingly, the reduction of IRP2 and TfR1 was not readily detectable in thermoneutral iBAT (Fig. 2*E*). Instead, we identified 1) upregulation of ER stress proteins, including CHOP, p-eIF2a, p-JNK, and p-38 (Fig. 2*F*); and 2) augmented autophagy, including the upregulation of autophagosome machinery (Beclin 1 and ATG5) and LC3II formation (Fig. 2*G*). These data imply that thermoneutral conditions induce ER stress in the iBAT and autophagic clearance of unnecessary mitochondria. Further, the iron released from the mitochondria seems to be sequestered into ferritin to avoid iron toxicity and exported out of iBAT.

Taken together, the changes in the thermogenic capacity of adipose tissues are correlated with the control of the IRP/IRE signaling and iron homeostasis.

### Differential regulation of IRPs during white and brown adipocyte differentiation

**Figure 3:**
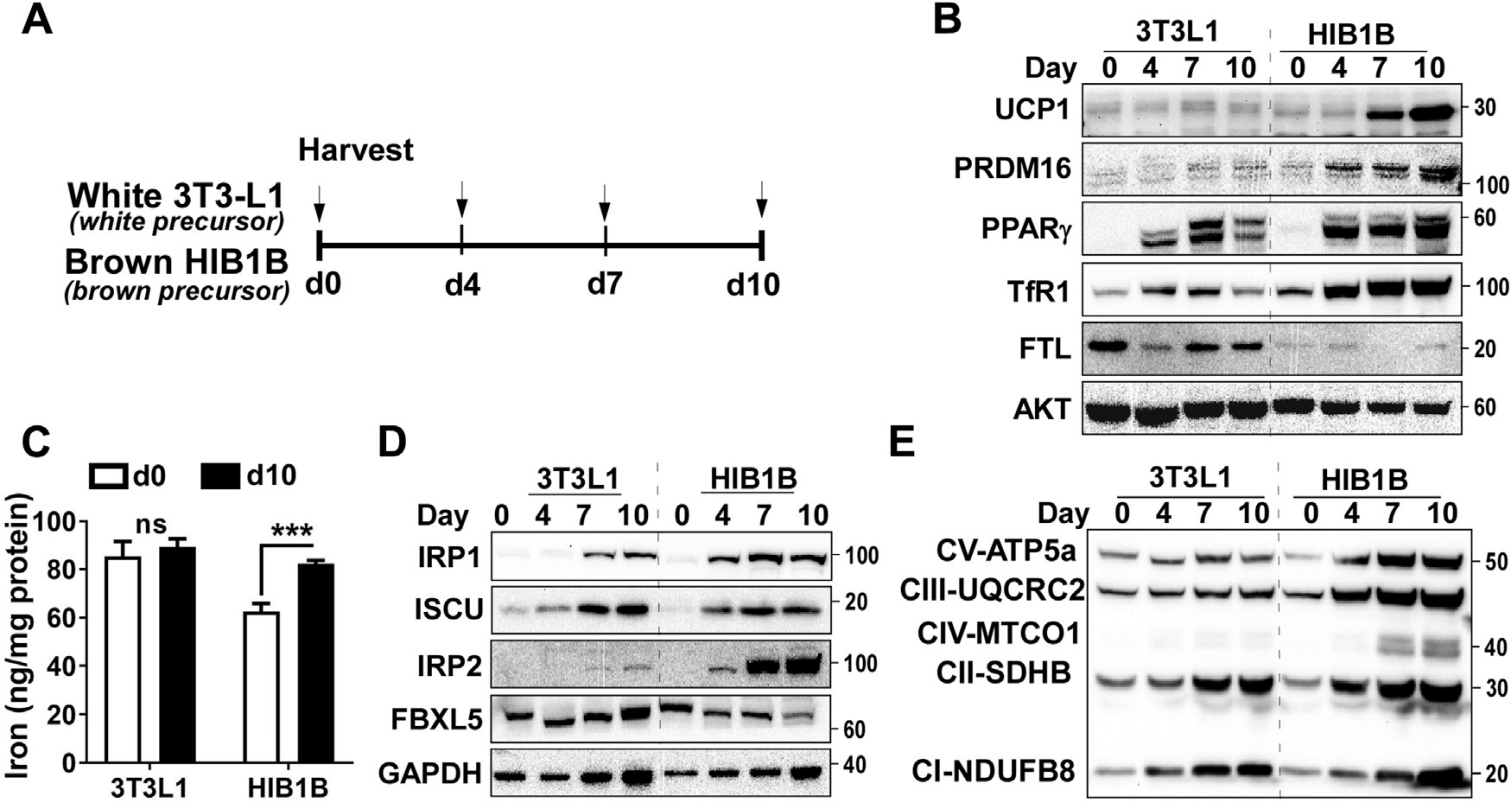
**Brown adipocytes had higher iron demands than white adipocytes during differentiation.** HIB1B brown preadipocytes and 3T3-L1 white preadipocytes are differentiated into brown and white adipocytes, respectively. *A*, experimental scheme. The kinetic changes in protein expression during white or brown differentiation by western blot analysis in *B*–*D* (n = 3), *B*, adipocyte markers (UCP1, PPARγ, PRDM16), transferrin receptor 1 (TfR1), ferritin light (FTL), and AKT (loading control), *C*, iron content of 3T3L1 and HIB1B at day 0 and day 10, *D*, IRP1 and 2 and their regulatory proteins of ISCU and FBXL5, andGAPDH (loading control), *E*, Mitochondrial respiratory chain components I to V. All western blot images were representative of two separate experiments. *Arrow* indicates the sample collection during adipocyte differentiation (days at 0, 4, 7, and 10). In *C*, ∗∗∗*p* < 0.001 by Student’s *t*-test (n = 5/group).

**Figure 4:**
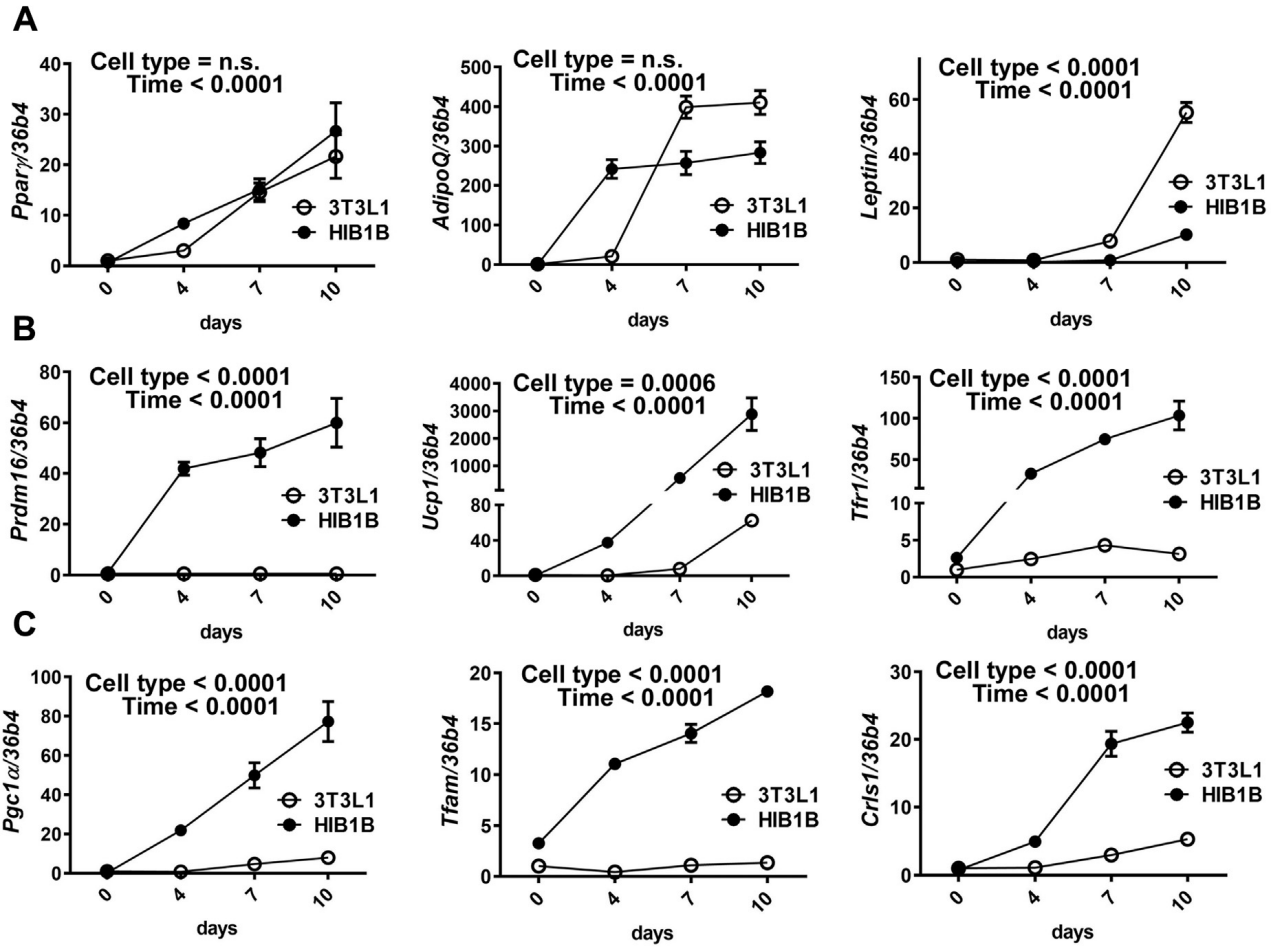
**The increase of transferrin receptor 1 was specific to brown adipocytes and paralleled mitochondrial biogenesis during differentiation.** HIB1B brown preadipocytes and 3T3-L1 white preadipocytes are differentiated into brown and white adipocytes, respectively. The kinetic changes (day 0, 4, 7, and 10) in mRNA levels during white or brown differentiation were measured qPCR (n = 3 for each time points/per group), *A*, mRNA levels of white adipocyte signature genes, *PPARγ*, *adiponectin* (*AdipoQ*), and *Leptin*, *B*, mRNA levels of brown adipocyte signature genes of *Prdam16* and *Ucp1* along with iron transporter *Tfr1*. *C*, mRNA levels of mitochondrial biogenesis related genes of *Pgc1α*, *Tfam*, and *Crls1*. In all data, *36b4* was the reference gene. All data are presented as mean ± SD. Two-way ANOVA analysis with two main factors of cell type (3T3-L1 or HIB1B) and differentiation time was used (*p* < 0.05).

Next, we questioned whether the regulation of IRP/IRE signaling and iron levels are the cell-autonomous mechanism that dictates white *versus* brown adipocyte differentiation. To address this question, 3T3-L1 and H1B1B cells, murine white and brown preadipocytes, respectively, were induced to differentiate (Fig. 3*A*), and we analyzed the kinetic changes of the proteins (Fig. 3) and mRNA (Fig. 4) related to iron regulation and mitochondrial biogenesis. Both 3T3-L1 and HIB1B cells were differentiated into lipid-laden mature adipocytes after 10 days of differentiation, showing the differentiation efficiency of ∼90% in both adipocytes (Fig. S1A). The expression of peroxisome proliferator-activated receptor-gamma (PPARγ), a key regulator in the complex process of adipocyte differentiation, was significantly increased over the differentiation process in both types of adipocytes. The protein levels of UCP1 and PRDM16, brown-specific markers, were markedly increased upon differentiation of brown adipocytes, which were negligible in white adipocytes. (Fig. 3*B*). In response to adipocyte differentiation, TfR1 expression continuously rises in brown adipocytes, while it showed only a transient increase of TfR1 in white adipocytes. In contrast to TfR1 expression, FTL was maintained remarkably low throughout the brown adipocyte differentiation, while FTL levels were kept high in white adipocytes with a transient decrease at day 4 (Fig. 3*B*). Consistently, the total iron content of 3T3L1 preadipocytes was higher than HIB1B preadipocytes, but it remained unchanged after 10 days of differentiation. However, the iron content of HIB1B cells was significantly increased after differentiation (62.5 *versus* 82.3 ng Fe/mg protein) (Fig. 3*C*). These results suggest that iron demands consistently rise for brown adipocyte differentiation, while iron demands are transiently increased only at the early differentiation process of white adipocytes, and the excess iron is stored as ferritin.

We also examined the differential regulation of IRP1 and IRP2 during brown *versus* white adipocyte differentiation. The abundance of IRP1 was gradually increased in both white and brown adipocytes, with a higher expression in brown than white adipocytes. Interestingly, the ISCU protein, a scaffold for synthesis of Fe-S cluster that masks IRP1-binding affinity to IRE, was higher in white adipocytes than in brown, suggesting that a bigger portion of IRP1 would be free to serve as RNA-binding protein in the brown adipocytes (Fig. 3*D*). In turn, IRP2 protein levels were barely detectable in white adipocytes upon full differentiation at day 10, while IRP2 accumulation showed an overt increase as early as 4 days postdifferentiation and continuously rose during brown adipocyte differentiation (Fig. 3*D*). IRP2, which does not bind a Fe-S cluster, is known to undergo targeted degradation by the iron-stabilized E3 ligase component FBXL5 (8, 9). This inverse relationship between FBXL5 and IRP2 was evident during adipocyte differentiation; the abundance of FBXL5 in 3T3-L1 white adipocytes correlated with a near absence of IRP2. Conversely, reduced FBXL5 protein in HIB1B adipocytes was enabled to escape FBXL5-mediated degradation and accumulate IRP2 within the cytoplasm (Fig. 3*D*). Consequently, the augmented iron influx and low iron storage during brown adipocytes (Fig. 3, *B* and *C*) are under the coordinated regulation of IRP1 and 2 (Fig. 3*D*). In parallel with an increase of iron import during brown adipocyte differentiation, there was a significant increase of mitochondrial respiratory chain proteins, suggesting that mitochondrial biogenesis is a key driving force for iron influx during brown adipocyte formation (Fig. 3*E*).

Next, we compared the kinetic changes of mRNA levels related to white- and brown adipocyte formation and mitochondrial biogenesis. The analysis was conducted with two-way ANOVA to determine the individual effect of cell type and time. All genes were significantly increased during the differentiation process in both white and brown adipocytes (time < 0.0001) (Fig. 4). The increase of *Pparγ* and adiponectin (*adipoQ*) shows no statistical significance between two cell types, although *adipoQ* levels reached a higher plateau in white adipocytes (Fig. 4*A*). On the contrary, leptin gene expression was significantly higher in white adipocyte differentiation than brown adipocytes, confirming that leptin is a white adipocyte marker (Fig. 4*A*). The increase of brown-specific markers, such as *Prdm16* and *Ucp1*, was significantly higher in brown adipocytes than white adipocytes (Fig. 4*B*), consistent with their protein expression (Fig. 3*B*). The mRNA expression levels of *Tfr1* were distinctively higher in brown adipocytes than in white adipocytes (Fig. 4*B*), which conforms with the posttranscriptional control by IRPs to stabilize their target mRNAs that possess IRE at the 3’UTR (6). The changes of mRNAs that are crucial for transcriptional regulation of mitochondrial biogenesis (*e.g.*, *Pgc1α* and *Tfam*) were dramatically higher in brown adipocytes than white adipocytes (Fig. 4*C*). Moreover, the increase of mRNA of cardiolipin synthase 1 (*Crls1*) that is a critical phospholipid for mitochondrial biogenesis was dramatically higher in concomitant with brown differentiation compared with white differentiation (Fig. 4*C*).

Collectively, these results indicate that brown adipocyte, but not white, differentiation is concurrent with the expansion of LIP (increased uptake but decreased storage). This brown-specific iron metabolism is under the control of IRPs to promote iron influx *via* TfR1 and to meet the demands for mitochondrial biogenesis.

### Iron deficiency interfered with cell-autonomous brown/beige adipocyte differentiation

**Figure 5:**
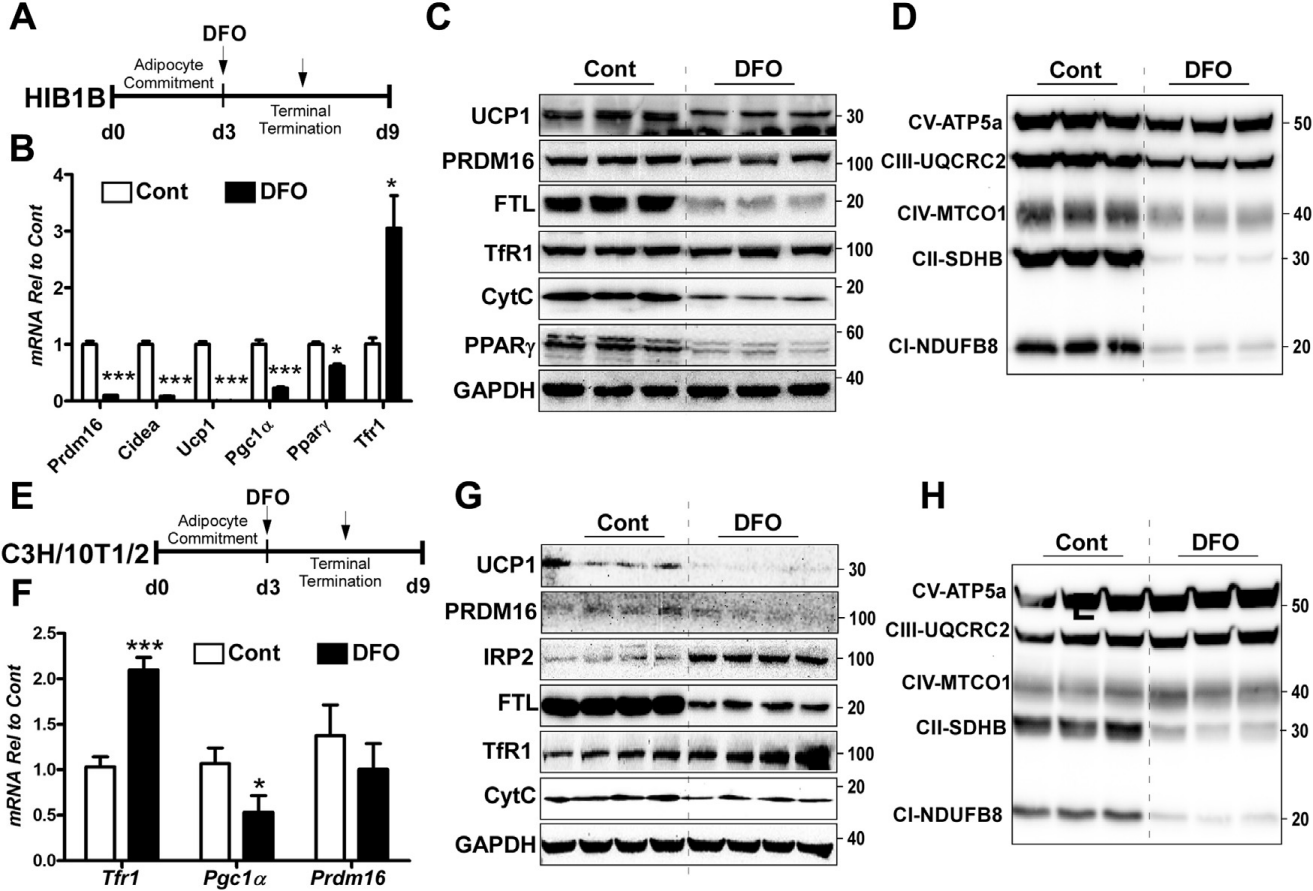
**Iron deficiency induced by chemical chelator interfered with brown and beige adipocyte differentiation.***A*, experimental scheme for DFO-induced iron deficiency in HIB1B-derived brown adipocytes (*B*–*D*), *B*, relative mRNA levels of *Prdm 16*, *Cidea*, *Ucp1*, *Pgc1α*, *Pparγ*, and *Tfr1* compared with control. *C*, protein expression of iron metabolism (UCP1, PRDM16, FTL, TfR1, PPARγ, and Cyt C). GAPDH used as a reference. *D*, mitochondrial respiratory chain components I to V, *E*, experimental scheme for DFO (100 nM)-induced iron deficiency in C3H/10T1/2-derived beige adipocytes (*F*–*H*), *F*, relative mRNA levels of *Prdm 16*, *Ucp1*, *Pgc1α*, and *Tfr1* compared with control, *G*, protein expression of iron metabolism (UCP1, PRDM16, IRP2, FTL, TfR1, and Cyt C). GAPDH is used as a reference. *H*, mitochondrial respiratory chain components I to V. *Arrow* indicates the addition of iron chelator DFO (100 nM). All data are presented as mean ± SD, ∗*p* < 0.05, ∗∗∗*p* < 0.001 by Student’s *t*-test compared with unstimulated control (n = 4 per group).

The cellular LIP reflects transitory and chelatable iron. We hypothesized that limiting the LIP compromises the cell-autonomous brown adipocyte differentiation. To test this hypothesis, we depleted the cellular iron by adding deferoxamine (DFO, 100 nM), a cell-permeable iron chelator, during HIB1B adipocyte differentiation (Fig. 5*A*). Chelation of intracellular iron right after adipocyte commitment step in HIB1B cells dampened brown adipocyte differentiation, including dramatic reductions in mRNA levels of *Prdm16*, *Pparγ*, *Pgc1α*, and *Ucp1* (Fig. 5*B*) and their protein levels (Fig. 5*C*). The decrease of LIP was confirmed by decreased iron storage protein ferritin (Fig. 5*C*) and augmented *Tfr1* mRNA (Fig. 5*B*) in the DFO-received cells compared with control. Accordingly, protein expression of mitochondrial oxidative phosphorylation (OxPhos) components I to V, as well as cytochrome C, was significantly reduced in response to DFO treatment (Fig. 5, *C* and *D*). Notably, the abundance of respiratory components I and II, which contain eight and three moieties of the Fe-S clusters, is more strongly affected by DFO treatment than components III and IV, which contain one moiety of Fe-S cluster or only heme irons (10). In contrast, component V, which has no iron, was minimally affected by DFO. These results are reminiscent of the iron limitation in myotubes by DFO treatment (11). These observations indicate that the chelation of labile iron significantly interferes with the entire differentiation process of brown adipocytes.

To determine the iron requirements during the beige differentiation, a similar experimental scheme was employed to C3H/10T1/2 cells, a pluripotent stem cell that is commonly used to obtain beige adipocytes (Fig. 5*E*). The DFO treatment in C3H/10T1/2 cells did not cause a significant difference in *Prdm16* mRNA levels (Fig. 5*F*), but PRDM16 protein levels were significantly lower than the control (Fig. 5*G*). DFO treatment significantly depleted the LIP, evidenced by the iron starvation responses displaying FTL suppression and TfR1 and IRP2 induction (Fig. 5*G*). Compared with the complete inhibition of mitochondrial proteins in HIB1B cells (Fig. 5*D*), DFO treatment only affects complex I and II and cytochrome C levels without changes in complexes III, IV, and V (Fig. 5*H*). This result reflects the higher iron requirement of CI and CII. More importantly, UCP1 expression was almost completely blunted upon DFO treatment. Collectively, limited iron availability during adipocyte differentiation significantly disrupts the brown and beige adipocyte differentiation program *per se*, a lesser degree to beige, and posed a detrimental impact on thermogenic mitochondrial biogenesis.

**Figure 6:**
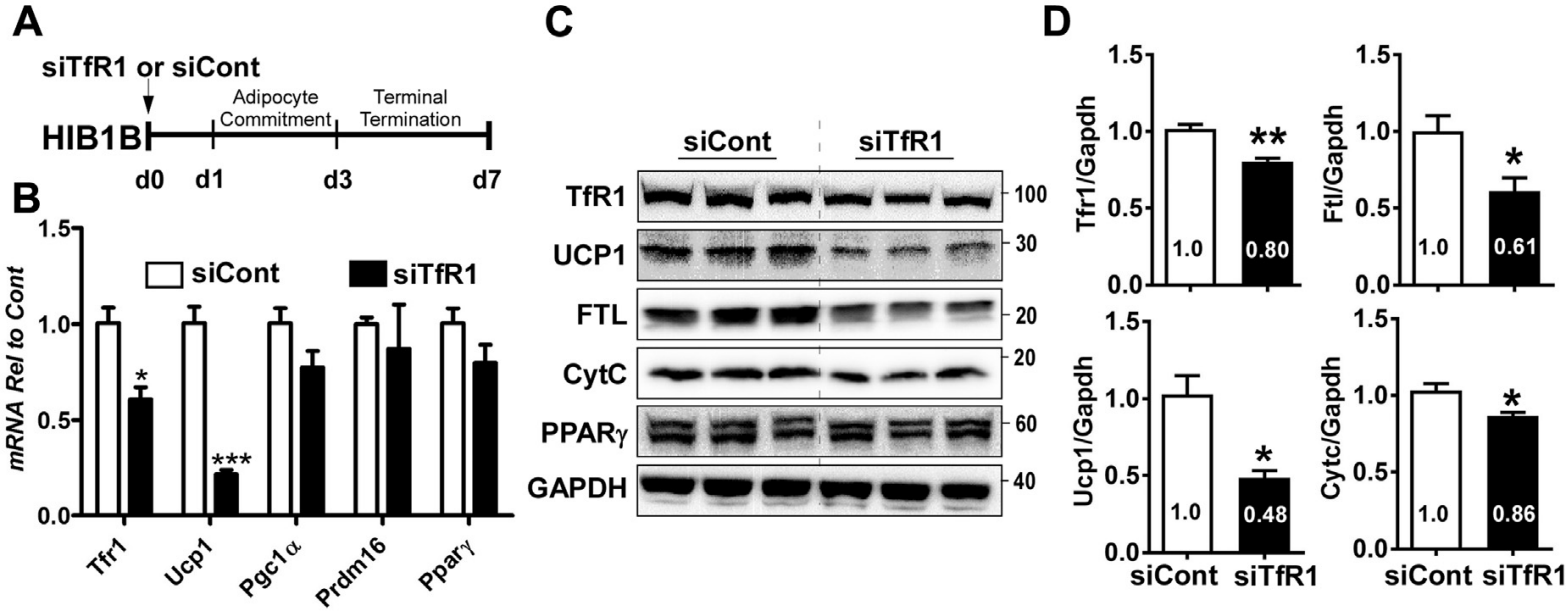
**Partial depletion of transferrin receptor 1 (TfR1) attenuated thermogenic potential in HIB1B brown adipocytes.***A*, experimental scheme HIB1B preadipocytes were transfected with either nontargeting siCont or siRNA targeting to TfR1 (siTfR1) and induced differentiation for 7 days. *B*, mRNA profiles of *Tfr1*, *Ucp1*, *Pgc1α*, *Prdm16*, and *Pparγ* upon 7 days differentiation, *C*, Protein expression of iron regulatory proteins (TfR1, FTL, and Cyt C) and brown adipocyte proteins of PPARγ and UCP1. GAPDH was used for loading control, *D*, quantified protein levels by Image J and normalized to siCont. In *B*, all data are presented as mean ± SD. ∗*p* < 0.05, ∗∗∗*p* < 0.001 by Student’s *t*-test with comparison to siCont (n = 4 per group). *Arrow* indicates the addition of siRNA (100 nM) to the cell.

To reduce LIP without causing serious effects on the adipocyte transcriptional program, we selectively depleted the transferrin receptor 1 by adding the siRNA targeting TfR1 (siTfR1) in comparison with the nontargeting siRNA (siCont) during HIB1B differentiation (Fig. 6*A*). The addition of siTfR1 resulted in a decrease of *Tfr1* mRNA levels by 40% (Fig. 6*B*) and its protein levels by ∼20% (Fig. 6, *C* and *D*). Reduction in ∼20% of TfR1 showed no significant challenge on adipocyte differentiation program, exhibiting similar levels of *Prdm16*, *Pparγ*, and *Pgc1α* compared with control (Fig. 6*B*). However, depletion of ∼20% of TfR1 expression resulted in ∼40% decrease of ferritin, indicating the reduced LIP size. Despite no noticeable differences in PPARγ (Fig. 6*C*) and mitochondrial respiratory chain proteins (Fig. S2), there was a significant reduction in UCP1 expression by 60% and a slight but significant reduction in cytochrome C by ∼15% (Fig. 6, *C* and *D*). Collectively, iron availability is a crucial determinant not only for brown adipocyte differentiation *per se* but also for competent thermogenic function.

### Iron uptake and compartmentalization into mitochondria occur autonomously during beige adipocyte differentiation

**Figure 7:**
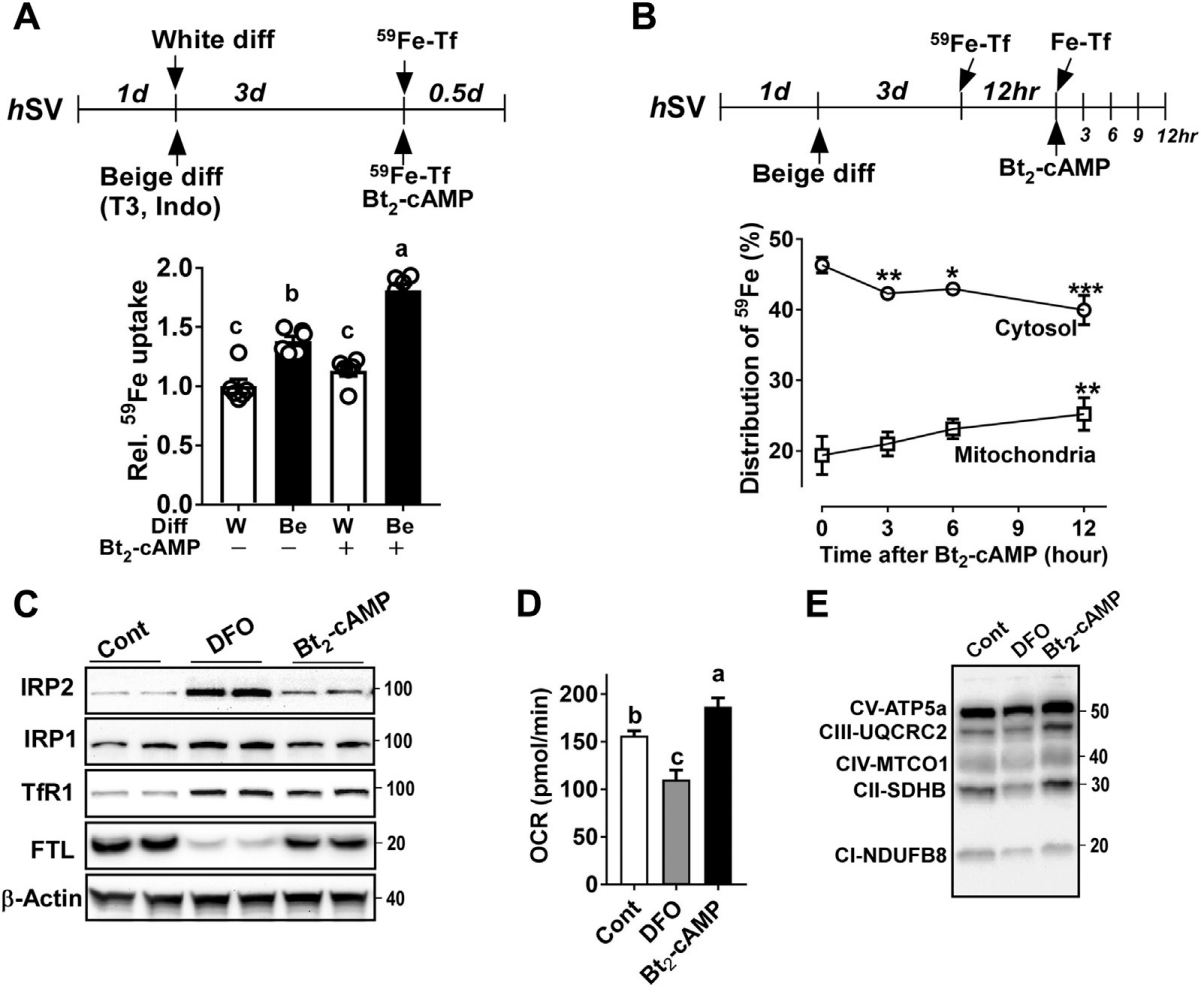
**The ADRB3-stimulated**^**59**^**Fe-Tf uptake was targeted to mitochondria in human SV cells derived beige, but not white, adipocytes.***A*, relative iron uptake using ^59^Fe-Tf in the newly differentiated beige or white adipocytes derived from uncommitted human SV cells in the presence (+) and absence (−) of Bt_2_-cAMP stimulation (n = 6/group), *B*, intracellular iron redistribution between cytosol and mitochondria during beige differentiation in response to Bt_2_-cAMP. ^59^Fe were tracked for 12 h with 3-h intervals (n = 6/each point), *C*, proteins profile of iron metabolism (IRP1, IRP2, TfR1, and FTL) in human beige adipocytes treated with either DFO (100 nM) or Bt2-cAMP (0.5 mM) compared with control, *D*, oxygen consumption rate (OCR), *E*, mitochondrial OxPhos proteins. In *A*, values not sharing a common letter differ significantly (*p* < 0.05) by one-way ANOVA. In *B*, ∗*p* < 0.05, ∗∗∗*p* < 0.001 by Student’s *t*-test compared with the values before Bt2-cAMP stimulation.

To confirm the role of IRP-dependent iron distribution in thermogenic adipocytes, we sought to compare the physical movement of iron and intracellular translocation during the white or beige adipocyte differentiation. To address this question, uncommitted human stromal vascular (*h*SV) cells were differentiated into either white or beige adipocytes (adding T3 and indomethacin) for 3 days, and then we measured iron uptake for 12 h by using ^59^Fe-Tf (transferrin bound ^59^Fe) as an iron tracer with or without cAMP analog. (Fig. 7*A*, *upper*). There was a substantial increase of ^59^Fe-Tf uptake after 3 days of incubation with beige differentiation medium compared with that of white differentiation (Fig. 7*A*, *lower*). Upon addition of dibutyryl cAMP (Bt_2_-cAMP), a cell-permeable nondegradable cAMP analog as key downstream signaling of β3-adrenergic receptor stimulation, the ^59^Fe-Tf uptake was further augmented by 1.8-fold in beige differentiation compared with white adipocytes (Fig. 7*A*). The intracellular distribution of ^59^Fe-iron between cytosol and mitochondria was tracked for 12 h. There was a stepwise decrease of cytosolic ^59^Fe, but a corresponding increase of mitochondrial ^59^Fe in a time-dependent manner (Fig. 7*B*), indicating the redistribution of cytosolic iron into mitochondria and the maintenance of low-cytosolic iron status as a consequence.

To obtain a better understanding of the relationship among iron demand, IRP activation, and adapted metabolic responses in adipocytes, we compared the iron-starvation responses induced by DFO (iron immobilization) *versus* Bt_2_-cAMP (adipocyte browning) stimulation. Similar to DFO responses in HIB1B and C3H/10T1/2 cells (Fig. 5), DFO treatment resulted in depletion of iron-storage protein FTL, stabilization of IRP2, and a robust increase of TfR1 expression in adipocytes as a collective sign of iron deficiency (Fig. 7*C*). However, DFO treatment decreased oxygen consumption rate (OCR) (Fig. 7*D*) as well as OxPhos protein expression (Fig. 7*E*), confirming the notion that adipocyte iron is required for mitochondrial function and respiratory protein transcription (7). The stimulation of Bt_2_-cAMP with human SV cells during beige differentiation triggered a substantial accumulation of IRP2, upregulation of TfR1, and slight reduction of FTL, which was comparable with DFO treatment (Fig. 7*C*). In contrast to DFO, Bt_2_-cAMP treatment resulted in increased OCR (Fig. 7*D*) and OxPhos protein expression compared with the control (Fig. 7*E*). These results suggest that IRP/IRE signaling should be coupled with external iron import and subsequent relocation into mitochondria for thermogenic mitochondrial development.

Based on these results, we propose a novel iron-regulatory network that β3-adrenergic receptor stimulation instigates cell-autonomous iron movement into mitochondria, which creates an iron-gradient between cytosol and mitochondria, thereby leading to subsequent IRP binding to IRE and iron uptake for thermogenic activation.

## Discussion

Iron is an essential but potentially hazardous biometal. Intracellular iron levels are tightly regulated in a narrow range to meet the lowest-sufficient level of cellular need, but to avoid the excess of harmful free iron (12). Over the last decade, numerous studies have demonstrated the diversity and flexibility of adipocyte subtypes in terms of cellular lineage and transcriptional regulation (5, 13, 14). However, it is largely unknown whether iron metabolism is different among adipocytes and how iron homeostasis is regulated to maintain the specificity of white, beige, or brown adipocytes. In this study, we demonstrated that the demands for iron are higher in brown/beige adipocytes than white adipocytes, which is correlated with mitochondrial biogenesis. Also, we showed that the restriction of LIP suppresses the cell-autonomous brown/beige differentiation. Collectively, our work suggests a novel role of iron as a crucial determinant for thermogenic adipocyte formation.

IRP/IRE interactions play a vital role in cellular iron homeostasis, including an increase in TfR1 to uptake iron and a decrease in ferritin to limit iron sequestration (6). Few studies have examined this iron-regulatory mechanism in adipocytes. Festa *et al.* (15) first demonstrated that iron-regulatory genes such as ferritin are upregulated during 3T3-L1 adipocyte differentiation to sequester the excess cytosolic iron into a safe form. Moreno *et al.* (16) reported the upregulation of IRP1 during 3T3-L1 differentiation and suggested that IRP1 is likely to serve as an aconitase rather than an RNA-binding protein. In supporting the essential role of aconitase in white adipocytes, the knockdown of IRP1 using lentivirus has shown to impair adipogenesis (16). Our results with 3T3-L1 adipocytes were consistent with these early observations in terms of IRP1 upregulation and negligible accumulation of IRP2 (Fig. 3*C*), presumably due to proteasomal degradation mediated by FBXL5, a ubiquitin ligase (E3) for iron-dependent degradation of IRP2 (8, 17). In contrast to these reports on iron metabolism in white adipocytes, surprisingly, few studies have been conducted on iron regulation during brown adipocytes differentiation. With a side-by-side comparison between white and brown adipocytes, we are the first to demonstrate that differentiation of HIB1B brown adipocytes is accompanied by 1) a dramatic increase of IPR2 as well as IRP1 (Fig. 3*D*) and 2) massive iron-starvation responses, including reduced ferritin and augmented TfR1 expression (Fig. 3*B*), indicating the enhanced IRP/IRE interaction. These results were validated *in vivo*, displaying the iron-deprivation responses in iBAT, but not in the eWAT or iWAT at the ambient temperature (Fig. 2*A*). In addition, we confirmed that IRP/IRE-binding affinity, by gel mobility shift assay, was higher in iBAT than in WAT, although we were unable to differentiate IRP1 from IRP2 (Fig. 1*B*). Meyron-Holtz *et al.* (18) demonstrated that IRP1 predominantly exists in cytosolic aconitase form, while IRP2 could selectively bind to IRE in response to iron starvation. Based on the complementary role of IRP1 and IRP2 (*i.e.*, deletion of IRP1 increases IRP2 and *vice versa*), neither of IRP1 nor IRP2 knockout mice have been reported for the congenital defects in adipose tissue (16). However, the thermogenic capacity and energy metabolism of these IRP1 or IRP2 knockout mice are yet to be determined. Based on our results and previous literature, we propose that both IRP1 and 2 are required for the development of brown adipocytes with maximal thermogenic function. In order to confirm this notion, the thermogenic function of adipose tissue should be investigated in IRP1 or IRP2 knockout mice as well as in animal models of adipocyte-specific ablation of IRP1 or IRP2 for future studies.

The rank order for iron levels in adipose tissue in comparison with other major organs is, eWAT ≈ iWAT << duodenum < iBAT < liver ≈ heart ≈ pancreas < spleen in normal physiological conditions (19). This result is consistent with our observation that iBAT is an organ requiring higher iron compared with other adipose depots. More importantly, we observed that the iron content of adipose tissue was modulated in response to external stimuli affecting thermogenic demands; 1) thermoneutral temperature attenuated the iron content in all depots (Fig. 2, *B* and *E*), and 2) β3-adrenoreceptor activation increased the iron content in iWAT concurrent with beige adipocyte formation within iWAT (Fig. 2*C*). The adjustment of iron content in adipocytes is correlated with mitochondria contents. The mitochondria from brown adipocytes have specific morphological characteristics (20); These mitochondria are apparently more numerous and bigger in size and contain more cristae than mitochondria in white adipocytes. The content of the heme cofactors of mitochondrial enzyme cytochrome oxidase (CIV) with a minor contribution of heme and Fe-S cluster of CII and CIII gives the tissue the brown macroscopic color (13). In addition, Fe/S clusters are also involved in mitochondrial function, including the TCA cycle, oxidative phosphorylation, and fatty acid oxidation (21). In our study, we interpreted the increased iron content during thermogenic activation in iWAT as a key factor for mitochondrial biogenesis (Fig. 2, *A*–*D*). It is well established that iron availability is a critical determinant for mitochondrial biogenesis by serving as a signaling molecule to regulate the transcription of mitochondrial proteins (11, 22). These studies provide us the rationale that dynamics of mitochondria during beige fat development should be coupled with an increase of IRP/IRE interaction for iron import. It is also supported by the fact that beige/brown precursors are distinctive in iron metabolism (5). Recently two publications by Li *et al.* and Qiu *et al.* demonstrated that blockage of iron influx to adipocytes by selective deletion of *Tfr1* in adipocytes reduces the mitochondrial biogenesis and thermogenic function (23, 24). These two studies conceptually overlap with what we observed in the current study in terms of the role of adipose tissue TfR1 and thermogenic regulation. Despite this, our study is unique in several points. We 1) provided the upstream regulation by IRPs binding to IRE in adipose tissue in a depot-specific manner and 2) revealed a previously unappreciated iron metabolism in thermogenic adipocytes, displaying redistribution of the cytosolic iron into mitochondria for mitochondrial biogenesis (Figs. 1 and 2).

Conversely, iron content was reduced in all fat depots at the thermoneutral setting (30 °C), where no physiological drive exists for heat production. It is notable that there were no significant differences in IRP2 and TfR1 expression levels in iBAT at 30 °C compared with 22 °C, despite an approximately 50% reduction of UCP1 and iron content (Fig. 2*E*). We speculate that the mechanism to remove the excess iron from iBAT is not a simple reversal of IRPs/IRE responses, and there is another layer of mechanism beyond the reversal of IRP/IRE signaling. In this study, we demonstrated the significant increase of ER-stress of iBAT (Fig. 2*F*), which seems to be coupled with autophagy activation (Fig. 2*G*), presumably for degradation of excess mitochondria. Supporting this notion, we have previously demonstrated that ER stress is a key mechanism behind autophagy-mediated mitochondrial removal in adipocytes (25). In addition, adipocyte whitening, a reversal process of beige fat (iWAT), is accompanied by clearance of mitochondria through an autophagy-mediated mechanism (3). It will be of interest to investigate whether the export of excess iron would be regulated by ferroportin, a transmembrane protein that serves as the only iron exporter (26). Nonetheless, thermoneutral conditions decreased the total iron content and increased the iron deposit into ferritin (Fig. 2*E*), suggesting the modulation of iron homeostasis adapting to a thermoneutral environment.

Adult humans possess significant amounts of brown/beige adipocytes. Since it was first identified in a decade ago (27, 28, 29), the metabolic benefits of brown/beige thermogenesis to attenuate the prevalence of obesity and metabolic complication have been extensively investigated (30, 31). Despite the identification of numerous factors that modulate adipose thermogenic function (32, 33), little is known about the relationship between adipose iron and thermogenic potential. Several studies report that iron deficiency attenuates thermogenesis in animal models and humans. Poor thermoregulatory capacity was observed in iron-deficient animals upon cold exposure, which was markedly restored by transfusion or iron treatment (34, 35). Iron-deficient humans failed to maintain their body temperature when they were exposed to cold water (36, 37). Iron supplementation recovered hypothermia in iron-deficient subjects under cool air exposure (38). These observations suggest that iron metabolism and thermogenesis are closely tied together. Besides iron deficiency anemia (IDA), obesity is linked with reduced iron uptake in the small intestine (39) and sequestration of iron into macrophages due to low-grade inflammation (40, 41, 42). Although there is continuous controversy whether IDA *per se* is a contributing factor for obesity, accumulating evidence suggests that deregulation of iron metabolism is involved in obesity-mediated inflammation and metabolic dysfunction (43, 44). We and others have demonstrated that high fat (HF)-diet-induced obesity is mediated with defective adipose tissue browning (25), which could be attributed to the inability to import iron into adipocytes due to failure of TfR1 expression in response to β3-adrenoceptor activation (24). In line with literature support, our results suggest that the immobility of iron in adipocytes resulted in defective brown/beige adipogenesis (Fig. 5). Moreover, only partial reduction of TfR1 (∼20%) can suppress UCP1 by 50% (Fig. 6*D*), indicating that adipose iron should be regarded as a critical determinant for development of brown/beige adipocytes and maximal thermogenic function.

Adipose tissue is composed of heterogeneous cells, and obesity is accompanied by an increased population of immune cells such as adipose tissue macrophages (ATM) (45). Emerging evidence suggests that obesity promotes iron accumulation in ATM, which significantly contributes to adipose tissue insulin resistance and metabolic complication (46, 47). Although we did not examine the competition of iron between adipocytes and ATM, it is reasonable to assume that reduced adipocyte browning in obesity (or beige fat differentiation) could stem from 1) the lack of available iron for beige precursor cells to differentiate into adipocytes due to sequestration of iron into ATM and 2) unable to import iron into beige adipocytes due to lack of iron transporter, TfR1. Therefore, the mechanism for “in-and-out of iron from adipocytes and ATM” requires further study toward developing a new strategy for enhancing adipose thermogenic capacity and energy expenditure.

In summary, we demonstrated that adipocyte iron metabolism is under the regulation of IRP/IRE regulatory system, which is a crucial characteristic to drive mitochondrial biogenesis and thermogenic function of depot-specific adipose tissue. Modulation of LIP in response to environmental stimuli is a critical factor for adjusting the mitochondrial oxidative potential of adipocytes, brown/beige development, and thermogenic capacity. Our study is the first to underscore the significance of IRPs/IRE interaction in the differentiation and maintenance of the thermogenic function of adipocytes. The iron clearance during adipocyte whitening and iron distribution between adipocytes and ATM warrant future research revealing the underlying mechanisms.

## Experimental procedures

### Animals

All protocols and procedures were approved by the Institutional Animal Care and Use Committee of the University of Nebraska-Lincoln and the University of Massachusetts-Amherst. To induce adipose tissue browning, C57BL/6 male mice were intraperitoneally injected daily with saline or β-3 adrenergic receptor agonist CL 316,243 (Santa Cruz Biotechnology, 1 mg/kg BW) for 5 days (n = 8 per group). To investigate the iron regulation at thermoneutral conditions, the mice were housed at either 22 °C (ambient) or 30 °C (thermoneutral) for 2 weeks (n = 8 per group). At necropsy, each mouse was fully perfused with 20 ml of ice-cold saline to remove the residual blood to avoid the potential contamination of iron from the blood. Three different depots of adipose tissues were collected from C57BL/6 mice: epididymal white adipose tissue (eWAT) as visceral fat, inguinal white adipose tissue (iWAT) as subcutaneous fat, and interscapular brown adipose tissue (iBAT) as constitutively active brown fat.

### Iron determination

For determination of the iron content, ∼50 mg of the adipose depot is digested in 70% nitric acid for 2 h at 75 °C. Total iron content in adipose tissues was analyzed by inductively coupled plasma mass spectrometry (ICP-MASS). The collected data are normalized by tissue weight. To avoid exogenous iron contamination, all glassware and plasticware were soaked in 10% nitric acid and washed with distilled water.

### Electrophoresis mobility shift assay (EMSA)

EMSA for IRP/IRE binding was performed based on the published protocol (48) with the following modification. The cytosolic fractions were prepared from eWAT, iWAT, and iBAT. Two micrograms of cytosolic protein was incubated with 16 μl of reaction mixture containing 5% glycerol, 0.2 units Super RNasin (Promega, N2511), 2 μg of yeast tRNA (Thermo Fisher, AM7119), 50 uM DTT, and 50 nM of IRDye700-IRE consensus RNA oligonucleotides in 10 mM Tris-HCl (pH 7.5), 10 mM MgCl_2_, and 100 mM KCl for 30 min at room temperature. The resulting reaction mixture was mixed with 2 μl of Orange loading dye (Li-Cor, P/N 927-10100), and load on a 4 to 12% acrylamide/TBE gel (Invitrogen) and run at 120 V for 3 h in the dark. The gel was scanned using Odyssey CLX System (Li-Cor). The RNA oligonucleotide consensus to IRE labeled with IRDye700 probe was synthesized from Integrated DNA Technology based on the published sequence; 5′-UCCUGCUUCAACAGUGCUUGGACGGAAC-3′ (49).

### Cell culture

3T3-L1 preadipocytes (ATCC, CRL173) were maintained with 10% bovine calf serum (BCS, GIBCO), and 2 days after reaching confluency, differentiation was induced with DMEM/H containing 10% FBS, 1 μg/ml of insulin, 390 ng/ml of dexamethasone, and 115 μg/ml of isobutyl-methylxanthine. After 2 days, media were changed to DMEM/H containing 10% FBS and 1 μg/ml of insulin and harvested on given days. The murine brown H1B1B preadipocytes were from Dr Johannes Klein (University of Lubeck, Lubeck, Germany) (50). HIB1B cells were differentiated as we described previously (51) and harvested on given days during differentiation. C3H/10T1/2 (ATCC CCL-226) cells, a pluripotent cell line derived from mouse embryos, display fibroblastic morphology and are functionally similar to mesenchymal stem cells (52). C3H/10T1/2 cells were included in this study as commonly used inducible beige adipocytes (53, 54). To induce beige differentiation, C3H/10T1/2 cells were maintained for 3 days in a medium supplemented with 5 μg/ml insulin, 0.5 mM 3-isobutyl-1-methylxanthine (IBMX, Sigma Aldrich), 2 μg/ml dexamethasone (Sigma Aldrich), 2 nM triiodothyronine (T3), 30 μM indomethacin, 5 μM rosiglitazone (Cayman Chemicals), 5 μM rosiglitazone (Cayman Chemicals), and then switched to medium supplemented with 5 μg/ml insulin for 6 days as described previously (53, 54).

Primary *h*SV cells were isolated from abdominal adipose tissue (subcutaneous fat) from adult females who had liposuction or abdominal plastic surgeries. To reduce the individual variation, pooled samples of 4 to 5 different human subjects were used for the experiments (Fig. 7). All protocols and procedures for human SV cell isolation were approved by the Institutional Review Board (IRB) at the University of Nebraska. For beige differentiation, confluent human SV cells were added to a differentiation mixture containing 500 nM insulin, 2 nM triiodothyronine (T3), 0.25 mM IBMX, 1 μM dexamethasone, 30 μM indomethacin (Indo), 1 μM rosiglitazone BRL49653, 17 μM pantothenate, and 33 μM biotin in 2% FBS for 3 days. For white differentiation, the same medium except for the absence of T3 and Indo was used for 3 days. After the initial commitment step, cells were changed to adipocyte sustaining medium that contains reduced insulin (100 nM) and the absence of IBMX before using for Tf-Fe uptake experiments (Fig. 7*A*). The experimental details for ^59^Fe-Tf uptake were found in the next session.

To chelate intracellular iron, either HIB1B or C3H/10T1/2 was incubated with 100 nM of DFO (Calbiochem) after 3 days of adipocyte commitment step and kept for 6 days throughout the terminal differentiation (Fig. 5, *A* and *E*)

### Knockdown of transferrin receptor 1 (TfR1)

siRNAs (On-Target plus, Dharmacon) targeting the mouse transferrin receptor 1 (*TfR1*) or nontargeting siRNA controls were transfected into HIB1B brown preadipocytes at a final concentration of 100 nM using DharmaFECT4 transfection reagent (GE Dharmacon, CO). Twenty-four hours posttransfection, HIB1B cells were washed and induced differentiation as described above.

### ^59^Fe-Tf uptake

To prepare ^59^Fe-Tf, a bioavailable form of iron bound with transferrin, 10 mg of apo-transferrin was incubated with 100 μCi of ^59^FeCl_3_ (PerkinElmer; Cat. #NEZ037500UC) in 100 mM of disodium nitrilotriacetate at room temperature for 1 h and then passed through a PD-10 column (GE Healthcare) that was equilibrated with 10 μM NaHCO_3_/0.25 M Tris-HCl buffer, pH 8.0 to remove unbounded ^59^Fe. ^59^Fe-Tf was eluted with 0.2 M sodium acetate buffer of pH 5.1 (flow rate of about 4 ml/min) and 5 ml volume fractions were collected and made a pool with fractions having absorbance at 280 nm. The ratio of A_465_/A_280_ was 0.046. The molar ratio of Fe/Tf was ∼1.6. For iron uptake experiments, hSV cells that are committed to either white or beige adipocytes were incubated with ^59^Fe-Tf (1 μCi/0.5 × 10^6^ cells) for 12 h. To determine the effects of β3-adrenoceptor stimulation, adipocytes were costimulated with dibutyryl cAMP (Bt_2_-cAMP, 0.5 mM) for 12 h (Fig. 7*A*). To determine the iron compartmentalization, hSV cells that are committed to beige adipocytes were incubated with ^59^Fe-Tf (1 μCi/0.5 × 10^6^ cells) for the first 12 h. Cells were washed thoroughly and nonradioactive beige differentiating medium with Bt_2_-cAMP (0.5 mM) for additional 12 h. Cells were harvested every 3 h and used for separation into a nuclear, mitochondrial, and cytosolic fraction (Fig. 7*B*). For the preparation of subfraction of cytosol and mitochondria, mitochondria/cytosol fractionation kit (Abcam 65320) was used according to the manufacturer’s instruction. ^59^Fe radioactivity was quantified from cytosol and mitochondria by gamma countering (Wizard 2, PerkinElmer).

### Western blot analysis

Total cell and tissue extract preparation and western blot procedures were conducted as we described previously (5). Each lane was loaded with 25 μg proteins of cell or adipose tissue lysates. The proteins were separated on 10 to 12% SDS-PAGE gels, transferred to polyvinyl difluoride membranes and polyvinylidene fluoride (PVDF) membrane, and immunoblotted with the relevant primary antibodies. Chemiluminescence from ECL solution (Western Lightning) was detected using an Odyssey FC Imaging System (Li-Cor) and analyzed with Image Studio Lite Software Ver 5.2.5 (Li-Cor). Details of antibody information are available in Table S1.

### qPCR

Total RNA was extracted from ∼100 mg of tissue or 0.5 million cells using TRIzolTM Reagent and treated with DNA-free DNA removal kit (ThermoFisher Scientific). Then the RNA was reverse-transcribed for cDNA synthesis (iScript, BioRad). Real-time PCR was carried out on a QuantStudio 6 Flex (Applied Biosystems) using SYBR Green (Fisher scientific). The relative gene expression was calculated based on the 2^−^ΔΔCT method with normalization of the raw Ct values by either *36b4* or *Gapdh*. For quantification of mitochondrial DNA content (mtDNA) in iWAT, DNA was isolated using DNAzol (Life Technologies). Quantitative PCR was performed in duplicate using mtDNA specific primer (16s rRNA) and nuclear DNA-specific primer (intron 9). Primer sequences are available in Table S2.

### Oxygen consumption rate (OCR)

To determine the mitochondrial respiration activities, the O_2_ concentration in the human adipocytes (derived from *h*SV cells) was measured using the XF24 extracellular flux analyzer (Seahorse), as we described previously (51).

### Statistics

All results are presented as means ± SD. The Student’s *t*-test was used to compare two independent groups, ∗*p* < 0.05, ∗∗*p* < 0.01, and ∗∗∗*p* < 0.001. Multigroup comparisons were performed by a one-way ANOVA followed by Tukey’s multiple comparisons. The depot-specific iron content at different temperature settings (22 °C and 30 °C) was analyzed by two-way ANOVA with Tukey’s multiple comparisons (Fig. 2*B*). The gene expression patterns of mitochondria development and iron transport during white and brown adipocyte differentiation (Fig. 4) were analyzed by two-way ANOVA with two main factors of cell type and differentiation time. All statistical analyses were performed using GraphPad Prism 7 (Version 7.03).

## Data availability

All data associated with this study are presented in the main text or supporting information.

## Supporting information

This article contains supporting information.

## Conflict of interest

The authors declare that they have no conflicts of interest with the contents of this article.
