# Supplementary material for: The thermogenic characteristics of adipocytes are dependent on the regulation of iron homeostasis
Source: J Biol Chem. 2021 Feb 23;296:100452. doi: 10.1016/j.jbc.2021.100452 (PMC8010711; doi:10.1016/j.jbc.2021.100452)
Supplement: Supplemental Figure S1–S2 and Tables S1–S2 [file mmc1.pdf]

Supporting Information

The thermogenic characteristics of adipocytes are dependent on the regulation of iron homeostasis

Jin-Seon Yook[1], Mikyoung You[1], Yongeun Kim[2], Mi Zhou[2], Zhenhua Liu[1], Young-Cheul Kim[1], Jaekwon Lee[3], and Soonkyu Chung[1,2§]

[1]Department of Nutrition and Health Sciences, University of Massachusetts, Amherst, MA
[2]Department of Nutrition and Health Sciences, University of Nebraska, Lincoln, NE
[3]Department of Biochemistry, University of Nebraska, Lincoln, NE
[§] Corresponding author: Soonkyu Chung Ph.D.

Table S1. List of primary antibodies

Antibody	Host	Dilution	Company	Catalog no.
UCP1	Rabbit	1:1000	Cell Signaling	14670
IRP1	Mouse	1:1000	Generously gifted by Dr. Rouault	
IRP2	Rabbit	1:1000	Cell Signaling	37135
TfR1	Mouse	1:250	Thermo Fisher Scientific	13-6800
FTL	Rabbit	1:1000	Abcam	ab69090
CytC	Rabbit	1:1000	Cell Signaling	4280
OxPhos	Mouse	1:250	Abcam	ab110413
FBXL5	Rabbit	1:200	Thermo Fisher Scientific	PA5-42296
ISCU	Mouse	1:100	Santa Cruz Biotechnology	sc-373694
Beclin 1	Mouse	1:1000	Santa Cruz Biotechnology	sc-48341
ATG5	Rabbit	1:1000	Cell signaling	12994
LC3II	Rabbit	1:1000	Cell signaling	4108
CHOP	Mouse	1:1000	Cell signaling	2895
p-eIF2a	Rabbit	1:1000	Cell signaling	9721
p-JNK	Mouse	1:2000	Cell signaling	9255
t-JNK	Rabbit	1:1000	Cell signaling	9252
p-p38	Rabbit	1:1000	Cell signaling	4511
PPARγ	Mouse	1:1000	Cell signaling	2443
PRDM16	Rabbit	1:1000	Santa Cruz Biotechnology	sc-130243
AKT	Rabbit	1:1000	Cell Signaling	9272
β-Actin	Rabbit	1:1000	Cell Signaling	4967
GAPDH	Mouse	1:1000	Santa Cruz Biotechnology	sc-137179

Table S2. Primer sequences for qPCR

Primer	Primer sequence	Size
Ucp1	F: 5′-AGGCTTCCAGTACCATTAGGT-3′<br>R: 5′-CTGAGTGAGGCAAAGCTGATTT-3′	133
Tfr1(Cd71)	F: 5′-AGCCAGATCAGCATTCTCTAAC-3′<br>R: 5′-TCTGCAGCCAGTTTCATCTC-3′	117
Prdm16	F: 5′- CAGCACGGTGAAGCCATTC-3′<br>R: 5′- GCGTGCATCCGCTTGTG-3′	87
PPARg	F: 5′- GGCGATCTTGACAGGAAAGAC-3′<br>R: 5′- CCCTTGAAAAATTCGGATGG-3′	151
Pgc1a	F: 5′- CCCTGCCATTGTTAAGACC-3′<br>R: 5′- TGCTGCTGTTCCTGTTTTC-3′	161
AdipoQ	F: 5′- ACAATGGCACACCAGGCCGT-3′<br>R: 5′- TGCCAGGGGTTCCGGGGAAG-3′	153
Leptin	F: 5′- CTTGCTCAAACCATGGTGATT-3′<br>R: 5′- GGCAGGCTTTCTATATGCTGA-3′	100
Tfam	F: 5′- GTCCATAGGCACCGTATTGC-3′<br>R: 5′- CCCATGCTGGAAAAACACTT-3′	204
Crls1	F: 5′- GGGCTACCTGATTCTTGAAGA-3′<br>R: 5′- GGCCCAGTTTCGAGCAATAA-3′	103
Gapdh	F: 5′- CATGGCCTTCCGTGTTCCTA-3′<br>R: 5′- GCGGCACGTCAGATCCA-3′	55
36b4	F: 5′-GGATCTGCTGCATCTGCTTG-3′<br>R: 5′-GGCGACCTGGAAGTCCAACT-3′	113

mtDNA genes	Primer sequence
16S rRNA	F: 5′-CCGCAAGGGAAAGATGAAAGAC-3′<br>R: 5′-TCGTTTGGTTTCGGGGTTTC-3′
Intron 9	F: 5′-GCCAGCCTCTCCTGATTTTAGTGT-3′<br>R: 5′-GGGAACACAAAAGACCTCTTCTGG-3′

Supporting Information

Figure S1

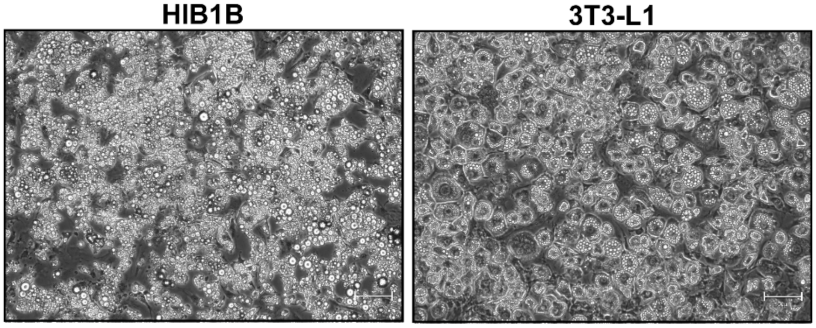

Figure S1. Microscopic images of lipid-laden brown (left) versus white (right) adipocytes. HIB1B and 3T3-L1 preadipocytes were induced to differentiate. Upon 10 days of differentiation, differentiation efficiency was similar displaying that ~ 90 % cells were converted into lipid-laden adipocytes.

Figure S2

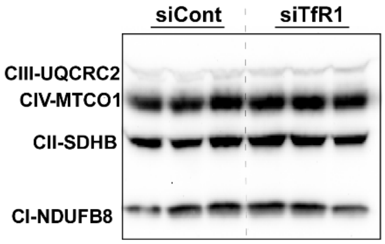

Figure S2. Partial depletion of transferrin receptor 1 (TfR1) showed no significant impact on OxPhos proteins. HIB1B preadipocytes were transfected with either non-targeting siCont or siRNA targeting to TfR1 (siTfR1) and induced differentiation for seven days. Proteins levels of OxPhos protein components were not different between two groups.
